# Efficacy and Safety of Shengxuening Combined with Conventional Iron Supplementation in the Treatment of Anemia during Pregnancy: A Systematic Review and Meta-analysis

**DOI:** 10.1155/2022/1773616

**Published:** 2022-10-13

**Authors:** Tianhang Li, Qian Wu, Yonggang Chen, Jili Zou, Enjing Zhang

**Affiliations:** Tongren Hospital of Wuhan University (Wuhan Third Hospital), Wuhan University of Science and Technology, Wuhan, China

## Abstract

**Background:**

To evaluate the efficacy and safety of Shengxuening combined with conventional iron supplementation in the treatment of anemia during pregnancy.

**Methods:**

Electronic searches of Embase, Web of Science, Cochrane Library, China Biomedical Literature Database (CBM), China Knowledge Network (CNKI), Wanfang database (Wanfang), and China Science and Technology Journal Database (VIP) were conducted. A randomized controlled trial of Shengxuening combined with conventional iron supplements for the treatment of anemia in pregnancy was included. The quality of the included trials was assessed using the Cochrane Handbook's Risk of Bias Assessment Tool (version 5.1.0), and data analysis was performed using RevMan 5.4 software and Stata 15.0 statistical software.

**Results:**

A total of 150 studies were detected and 17 studies with a total of 1741 cases were finally included. Meta-analysis results showed that the effectiveness of Shengxuening combined with conventional iron supplementation was significantly better than that of conventional iron supplementation alone, and the adverse effects were significantly reduced compared with that of iron supplementation alone, and various anemia indicators such as Hb, RBC, MCV, and MCHC and iron metabolism indicators such as SI and TSAT were also significantly increased.

**Conclusion:**

The efficacy of the combination of Shengxuening and conventional iron supplementation in the treatment of anemia in pregnancy is better than that of conventional iron supplementation alone, and the adverse effects in the experimental group are much less than those in the control group, but the quality of the included studies is not high, and more high-quality randomized controlled trials are needed for further validation.

## 1. Introduction

Anemia is a relatively common comorbidity during pregnancy. Due to the increase in blood volume during pregnancy and the increase in plasma more than the increase in red blood cells, the blood is diluted, also known as “physiological anemia” [1]. Many studies have shown that pregnant women with anemia during pregnancy are at higher risk for postpartum depression [2]. Anemia during pregnancy can be harmful to both the mother and the fetus to some extent. Anemic pregnant women are less able to tolerate childbirth, surgery, and are prone to hemorrhagic shock. In contrast, moderate-to-severe anemia causes the placenta to be inadequate to meet the needs of the growing fetus, which can lead to fetal birth restriction and preterm delivery. Iron deficiency anemia is the most common. For patients with mild-to-moderate anemia, clinical advice is to take oral iron supplements as well as to improve the diet structure and eat more iron-rich foods. Patients with severe anemia are recommended to be treated with oral iron or intravenous iron, with small and multiple infusions of concentrated red blood cells as needed. The commonly used clinical oral iron agents are ferrous succinate, iron polysaccharide, and Shengxuening. Commonly used oral iron supplements are ferrous succinate, iron polysaccharide, iron gluconate, iron sulfate, and hematopoietin. Conventional iron such as ferrous succinate is prone to gastrointestinal adverse effects such as nausea and vomiting, constipation, and diarrhea [3]. Shengxuening is an extract of dried feces of silkworm moths, the main component of which is sodium ferric chlorophyllin, which is similar in structure to heme and can directly participate in the hematopoietic process with low incidence of adverse reactions [4]. In recent years, the combination of Chinese patent medicine and Western medicine iron in the treatment of anemia has become a common practice, and clinical reports emerge one after another. However, the sample size of the current combination drug study is small and the evidence is insufficient. It is necessary for us to make a systematic review of the related clinical studies of the combination drug, so as to provide more bases for clinical decision-making.

## 2. Methods

Inclusion criteria are as follows: (1) included in the study from January 1, 2000 to March 1, 2022; (2) type of included studies: clinical randomized controlled trials (RCT); (3) patients: patients who were clearly diagnosed with iron deficiency anemia in pregnancy, in accordance with the guidelines for the diagnosis and management of iron deficiency and iron deficiency anemia in pregnancy; there were no restrictions on age, gender, or primary disease; (4) intervention: experimental group: treatment with Shengxuening in combination with conventional iron supplementation; control group: treatment with iron alone. Both groups received the same course of treatment and were treated with dietary modification and discontinuation of other medications and symptomatic support during the experimental period. (5) outcomes: I. Clinical efficiency (including cured, effective, and ineffective): cured: anemia symptoms effectively cured, and all indicators are normal; effective: anemia symptoms were relieved and indicators showed improvement; and ineffective: no change in anemia symptoms. Total effective = Cured + Effective; II. Anemia indicators: hemoglobin (Hb)、red blood cell count (RBC), mean red blood cell volume (MCV), average red blood cell hemoglobin concentration (MCHC), transfer iron saturation (TSAT), serum iron (SI); III. Incidence of adverse reactions; (6) exclusion criteria are as follows: nonclinical randomized controlled trials; the control group did not meet the entry criteria; uncritical experimental design, such as lack of informed consent in the literature; (7) search strategy: the keywords are “Shengxuening” or “Shengxuening tablets” or “Faces bombycis extract” or “Silkworm Sand” and “iron deficiency anemia” or “anemia” and “pregnancy” or “pregnancy” or “perinatal period”. We searched Embase, Web of Science, Cochrane Library, China Biomedical Literature Database (CBM), China National Knowledge Infrastructure Database (CNKI), Wanfang Database (Wanfang), and China Science and Technology Journal Database (VIP).

### 2.1. Literature Screening and Quality Assessment

Two authors independently and separately screened the literature according to the inclusion and exclusion criteria, and differences were further confirmed by a third party. The quality of individuals included in the study was assessed on the basis of the Cochrane Handbook's Risk of Bias Assessment Tool (version 5.1.0), including random sequence generation, allocation concealment, blinding of participants and personnel, blinding of outcome assessment, incomplete outcome data, selective reporting, and other biases. The deviation map risk and deviation summary risk assessments were performed using RevMan 5.4 software.

### 2.2. Data Extraction

General information about the eligibility study including first author's name, baseline, sample size, gender, age, intervention, treatment course, outcome, and adverse events was collected.

### 2.3. Statistical Analysis

RevMan 5.4 and Stata 15.0 statistical software were used for meta-analysis. I^2^ was the evaluation index of between-group heterogeneity. I^2^ ≤ 50% indicated that the difference between groups was small, and the fixed-effect model was chosen; I^2^>50% indicated that the difference between groups was large, and the random-effect model was chosen. Sensitivity analysis was used to find the sources of heterogeneity by excluding literature one by one and subgroup analysis.

## 3. Results

### 3.1. Search Results

**Figure 1:**
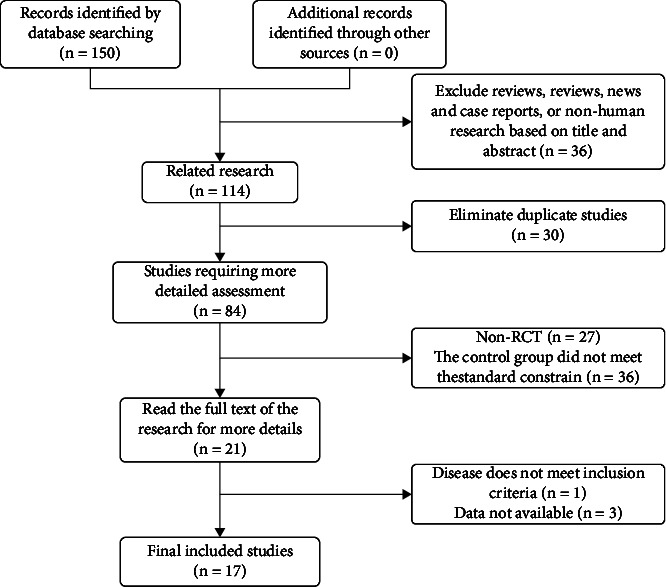
Flow diagram of searching for eligible studies.

By searching Embase (*n* = 1), Web of Science (*n* = 0), Cochrane Library (*n* = 1), CBM (*n* = 12), CNKI (*n* = 60), Wanfang (*n* = 40), and VIP (*n* = 36), a total of 150 studies were retrieved. Based on the inclusion and exclusion criteria, 17 studies were included in the final analysis. The procedure for selecting eligible studies is presented in Figure 1.

### 3.2. Including the Characteristics of the Study

Summarized baseline characteristics including studies are shown in Table 1. There were 17 RCTs (*n* = 1741). All studies were conducted in China, and all studies were published in Chinese. Treatment durations ranged from four weeks to twelve weeks.

### 3.3. Risk of Bias Assessment

**Figure 2:**
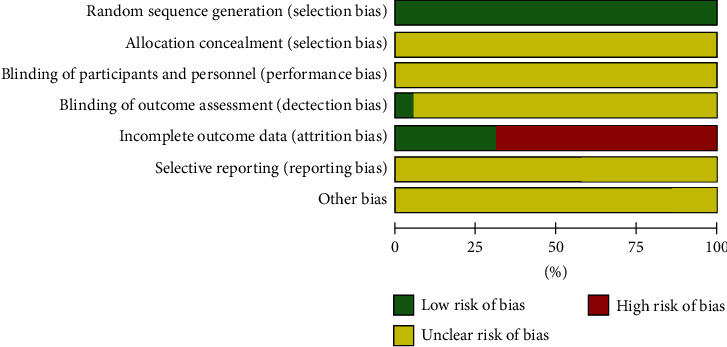
Risk of bias graph.

**Figure 3:**
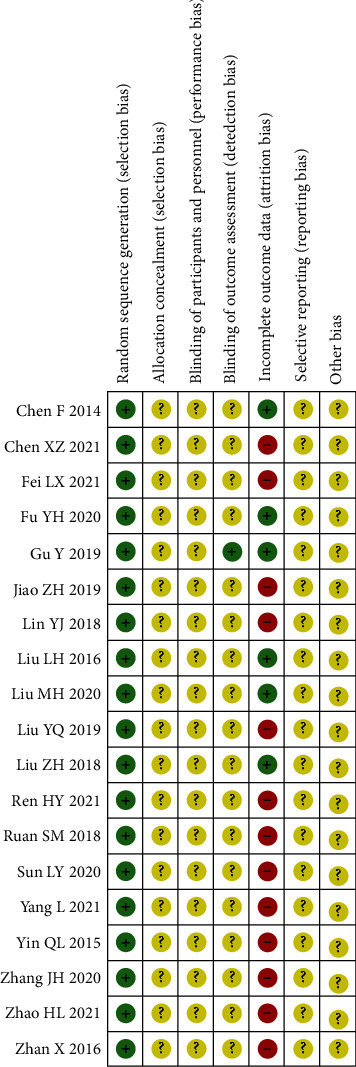
Risk of bias summary.

Randomization tables were used for randomization in 12 studies, while the remaining 3 trials only mentioned “randomization” and the specific method was not reported. None of the studies mentioned allocation concealment and blinding. There was no information on incomplete outcome data, selective reporting, and other biases. The risk of bias assessment regarding the quality of the methods is presented in Figures 2 and 3.

### 3.4. Meta-Result Analysis

#### 3.4.1. Effectiveness

**Figure 4:**
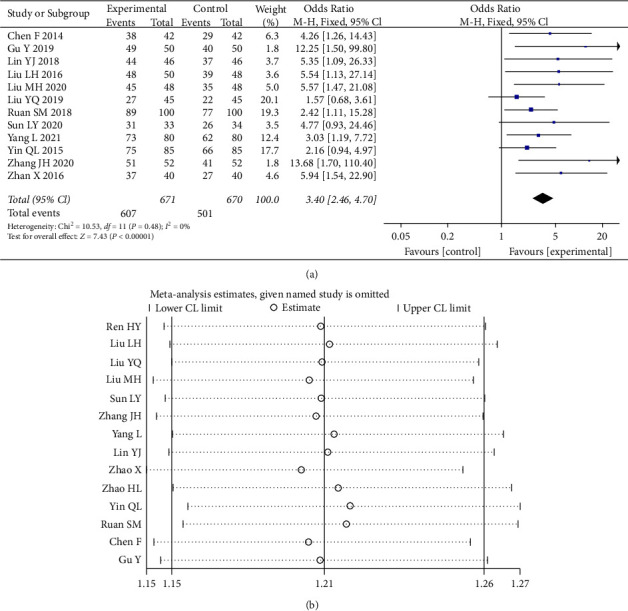
Forest plot and sensitivity analysis of meta-analysis of effectiveness.

As can be seen from Figure 4, 13 publications were included [5, 7–14, 17–21]. The total effective rate in the experimental group was 90.46%. The total effective rate in the control group was 74.78%, which was statistically significant (*p* < 0.00001). The heterogeneity test I^2^ = 0% indicated that the heterogeneity between the combined studies was small, and the fixed-effects model was selected with a 95% CI interval of (2.46, 4.70). Sensitivity analyses showed no significant change in effect size after excluding each study. As shown in the figure, the clinical efficacy of the experimental group was significantly better than that of the control group.

#### 3.4.2. Adverse Reactions

**Figure 5:**
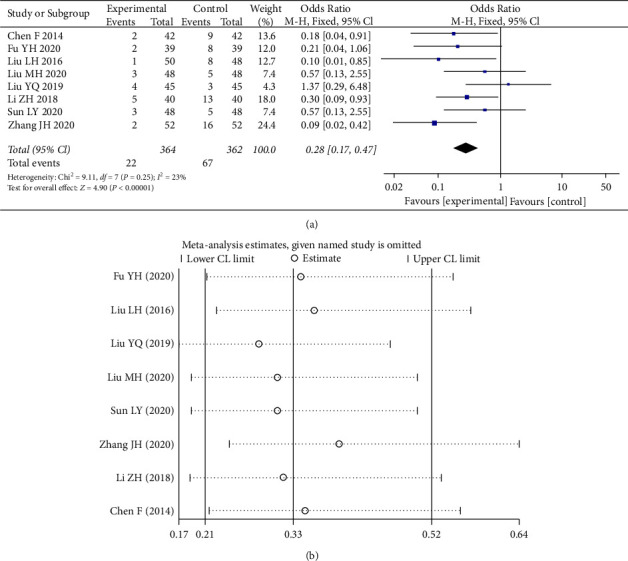
Forest plot and sensitivity analysis of meta-analysis of adverse reactions.

As can be seen from Figure 5, a total of eight papers were included [6, 8, 9, 11–13, 19, 20]. The incidence of adverse reactions in the experimental group was 6.04%. The incidence of adverse reactions in the control group was 18.5%, which was statistically significant (*p* < 0.00001). The heterogeneity test I^2^ = 23% indicated a small heterogeneity between the combined studies, and a fixed effects model was selected with a 95% CI interval of (0.17, 0.47). Sensitivity analyses showed no significant change in effect size after excluding each study. Among these studies 2, 4, 7, 9, 15, and 16 mentioned gastrointestinal symptoms such as nausea and vomiting, epigastric discomfort, and constipation. Meta-analysis results showed that the adverse effects of Shengxuening in combination with iron supplementation were significantly lower than those of iron supplementation alone.

#### 3.4.3. Hemoglobin (Hb)

**Figure 6:**
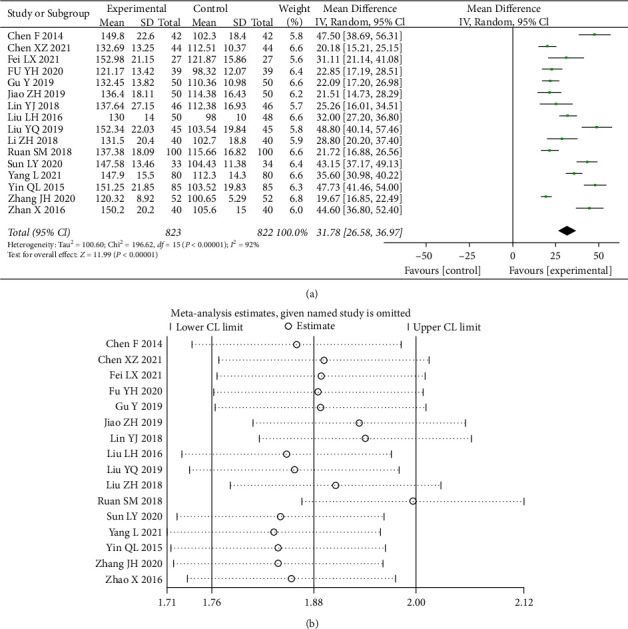
Forest plot and sensitivity analysis of meta-analysis of hemoglobin.

A total of 17 studies [5–11, 13–21] were counted for Hb, and the test of heterogeneity (I^2^ = 92%) indicated significant heterogeneity between the data of the included studies, choosing a random-effects model with a 95% CI (26.58, 36.97) with a statistically significant difference (*p* < 0.00001) (Figure 6). A sensitivity analysis reveals the source of heterogeneity. As can be seen from the graph, the hemoglobin levels were significantly higher in the combination group than in the control group.

#### 3.4.4. Red Blood Cell Count (RBC)

**Figure 7:**
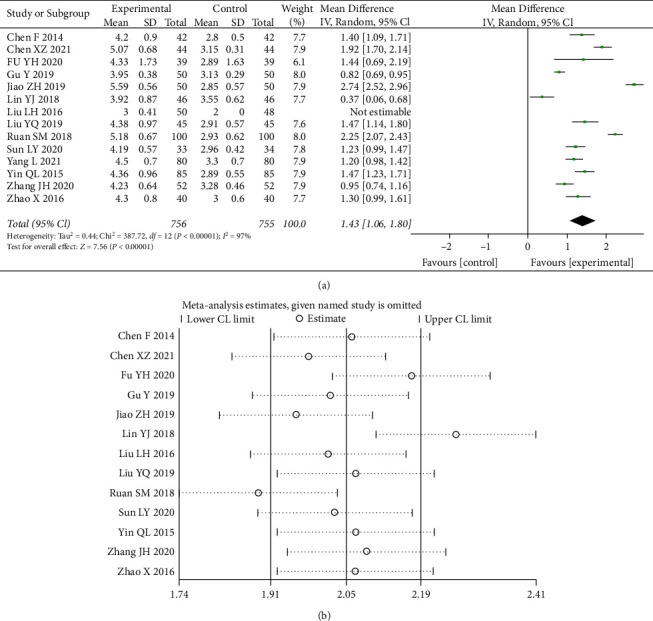
Forest plot and sensitivity analysis of meta-analysis of red blood cell count.

A total of 14 studies [5, 8–11, 13–17, 19–21] counted data from RBC (Figure 7). Heterogeneity tests (I^2^ = 97%) indicated significant heterogeneity between the data of the included studies. A random-effects model with 95% CI (1.06, 1.80) was chosen and was statistically different (*p* < 0.00001). It can be seen that the combination of drugs significantly increased the number of red blood cells compared to the control group.

#### 3.4.5. Mean Red Blood Cell Volume (MCV)

**Figure 8:**
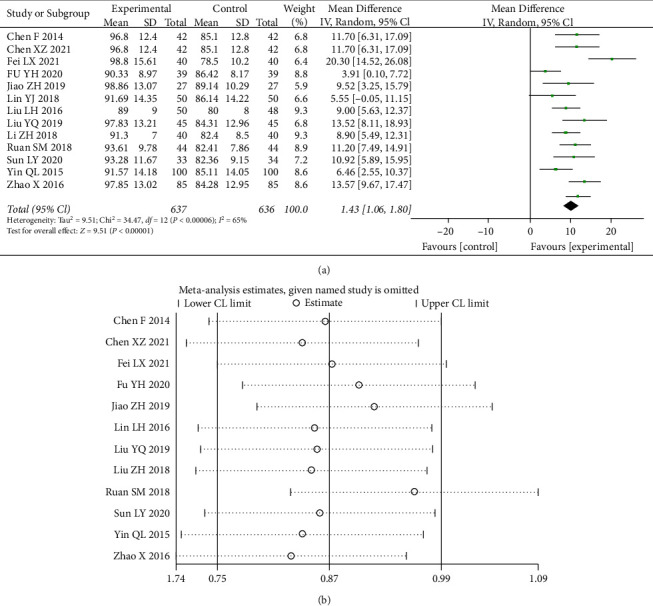
Forest plot and sensitivity analysis of meta-analysis of mean red blood cell volume.

A total of 13 studies [6, 9, 11–21] counted data on MCV, and the test of heterogeneity (I^2^ = 65%) indicated statistically significant heterogeneity between statistics, choosing a random-effects model with a 95% CI(8.14, 12.37) (*p* < 0.00001) (Figure 8). It can be seen that the change in MCV was significantly better in the combination group than in the control group.

#### 3.4.6. Mean Red Blood Cell Hemoglobin Concentration (MCHC)

**Figure 9:**
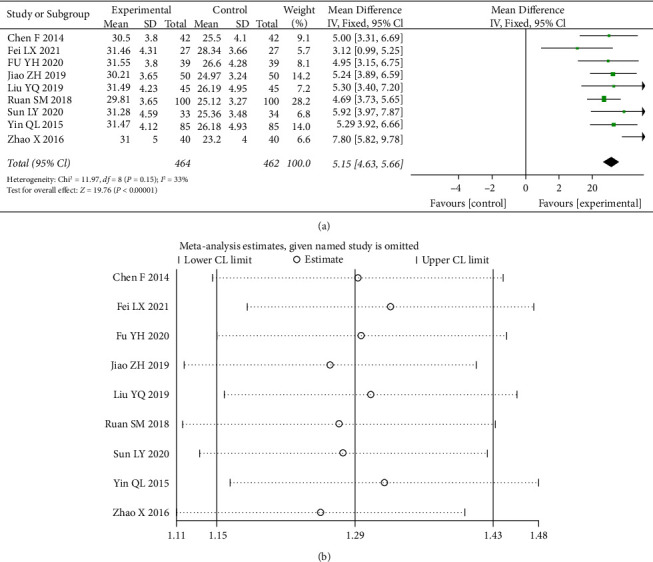
Forest plot and sensitivity analysis of meta-analysis of mean red blood cell hemoglobin concentration.

A total of 10 studies [9, 11, 13, 16, 18, 19, 21] counted data from MCHC, and the test of heterogeneity (I^2^ = 33%) indicated a small heterogeneity between statistics, with a statistically significant difference (*p* < 0.00001) by choosing a fixed-effects model with a 95% CI confidence interval (4.63, 5.66) (Figure 9).

#### 3.4.7. Transfer Iron Saturation (TSAT)

**Figure 10:**
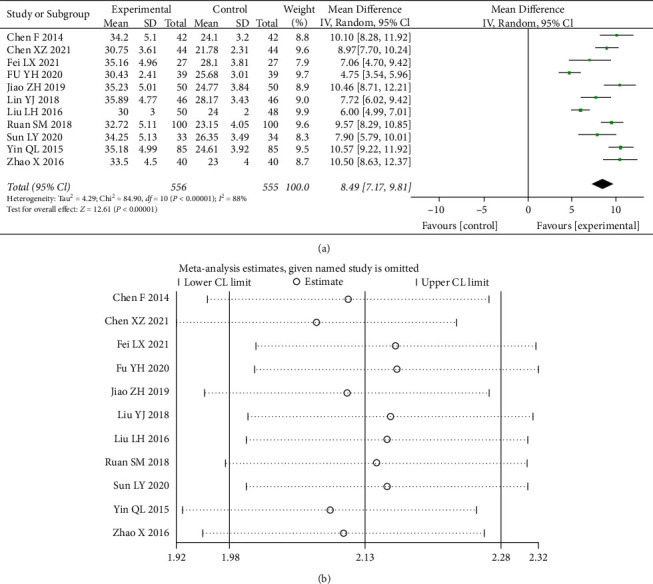
Forest plot and sensitivity analysis of meta-analysis of transfer iron saturation.

A total of 14 studies [5, 9–11, 13–21] counted data from the TSAT, and the heterogeneity test (I^2^ = 88%) indicated significant heterogeneity between the included data, choosing a random-effects model. 95% CI (7.17, 9.81) was statistically different (*p* < 0.00001) (Figure 10). It can be seen that the improvement in TSAT, an indicator of iron metabolism, was significantly better in the combination drug group than in the control group.

#### 3.4.8. Serum Iron (SI)

**Figure 11:**
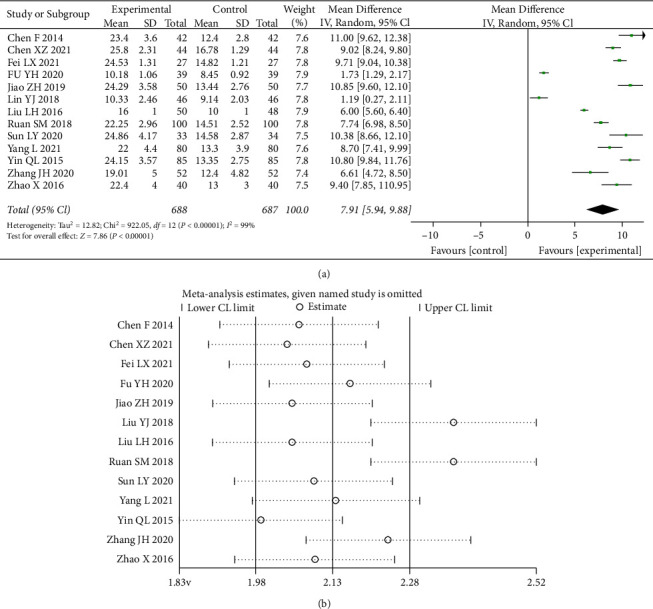
Forest plot and sensitivity analysis of meta-analysis of serum iron.

A total of 15 studies [7–11, 13–21] counted data for SI, and the test of heterogeneity (I^2^ = 68%) indicated statistically significant heterogeneity between the data of the included studies, choosing a random-effects model with 95% CI (5.94, 9.88) (*p* < 0.00001) (Figure 11). A sensitivity analysis reveals the source of heterogeneity. As shown in the figure, the improvement in the iron metabolism index SI was significantly higher in the combination group than in the control group.

In conclusion, the anemia-related indexes such as Hb, RBC, MCV, and MCHC were significantly improved when Shengxuening was combined with iron-supplementing drugs compared with iron-supplementing drugs alone, and the iron metabolism indexes such as SI and TSAT were also significantly increased. This indicates that the combination of Shengxuening and iron-supplementing drugs has better clinical effectiveness.

## 4. Discussion

Current studies related to anemia in pregnancy have focused on comparative studies of different iron treatments, for example, researchers have found similar performance of oral iron, with parenteral preparations being the most effective [22]. New oral iron formulations have also shown promising clinical efficacy and reduced incidence of adverse reactions, such as iron bisglycinate and liposomal iron formulations, which have high gastrointestinal absorption and bioavailability, as well as a lower incidence of adverse reactions than that of conventional oral iron formulations [23]. In this special group of patients with anemia during pregnancy, the medication should be considered in terms of patient compliance and tolerability. Based on clinical considerations, we found that the combination of proprietary Chinese medicine with iron therapy can significantly promote the recovery of iron metabolism and blood parameters with a lower incidence of adverse effects than iron alone.

The main component of Shengxuening is sodium iron chlorophyll. Its structure is similar to human heme. After oral administration, it can directly participate in the synthesis of human blood [24]. Its absorption is not competitively inhibited by other divalent metal ions, does not produce free iron, has no stimulating effect on the gastrointestinal tract, and does not cause iron accumulation poisoning with long-term administration, which has unique clinical advantages [6].

In a study by Sumin Li, it was shown that the combination of Shengxuening and ferrous succinate tablets had better efficacy, shorter recovery time of anemia index, and less incidence of adverse effects than ferrous succinate tablets alone [25]. Chunhua Ji's study showed more significant efficacy and less incidence of adverse reactions with Shengxuening combined with polysaccharide iron complexes [26]. These studies share the findings of the present study that the combined action of Shengxuening with different iron supplement preparations provides a stable and effective source of iron intake to the hematopoietic system and may reduce to some extent the dose of conventional iron supplements taken to alleviate their side effects.

At the same time, basic animal experiments have shown that Shengxuening can effectively promote the proliferation of bone marrow erythroid progenitors and granulocyte-macrophage progenitors in mice; increase the percentage of peripheral blood reticulocytes; promote the recovery of erythrocytes, hemoglobin, and reticulocytes in hemorrhagic rats; and increase the serum iron content and transferrin saturation [27].

On the other hand, iron regulators are important iron homeostasis-regulating hormones that function to regulate the imbalance of iron homeostasis in the body [28]. Under normal physiological conditions, the expression of iron-regulated hormones in the body shows a negative feedback relationship with the level of iron in the body, and when iron-regulated hormones are overexpressed, it leads to insufficient iron in the body and subsequently to anemia. It was found that Shengxuening inhibited iron-regulatory protein expression by blocking the JAK2/STAT3 signaling pathway [29]. At the same time, Shengxuening upregulates iron regulatory protein expression, increases the binding of iron regulatory proteins and iron response elements, and promotes the release of iron from the liver to supply the body's needs [30].

It has been found that Shengxuening attenuates myelosuppression, increases bone marrow CFU-E and CFU-GM production, increases reticulocyte percentage, and restores hematopoiesis in a hemorrhagic anemia model through the stem cell factor-mediated JAK2/STAT3 signaling pathway [20, 31].

In summary, Shengxuening agents further improve the symptoms of anemia by promoting the bone marrow to perform its hematopoietic function, facilitating the release of iron from the liver and providing an effective source of iron for the hematopoietic system.

From the meta-analysis, it can be seen that the combined application improved the efficacy of conventional iron supplementation preparations and shortened the dosing time; thus, reducing gastrointestinal reactions such as nausea and vomiting, decreasing the incidence of adverse reactions and improving patient compliance, which is beneficial for use in patients during pregnancy.

## 5. Method Quality Assessment

Selection bias can be prevented by using randomization. All of our selected studies were randomized controlled trials, and only three of them mentioned “randomization” only, without mentioning the specific method. The remaining studies all used random number tables. Allocation hiding also prevents selection bias; however, none of the studies we selected described whether allocation hiding was used.

None of the selected studies mentioned whether blinding was used.

### 5.1. Limitations

This paper has some potential limitations. (1) Language bias: the languages included in the study were Chinese and English, but the final included literature was all in Chinese, and studies in languages other than these two were not included and studied. (2) Publication bias: articles with positive results are more likely to be published and retrieved. (3) Quality studies: the quality of the included studies was not high and the baseline data were more ambiguous, which may have some influence on the conclusions of the study results. (4) Heterogeneity of included studies: after performing sensitivity analyses, excluding literature and subgroup analyses on a case-by-case basis, some indicators could not be excluded from heterogeneity, and the source of heterogeneity was unclear and may affect the conclusions of the study results. (5) Selection of outcome indicators: only some anemia indicators and some iron metabolism indicators were selected in this paper, which did not completely cover all clinical outcome indicators and may affect the evaluation of the clinical efficacy of the combination therapy of Shengxuening. (6) The evaluation of the quality of the included literature varies from person to person and is more subjective. (7) Ongoing studies are not included. (8) The control group included in the study was small and did not cover all clinically used oral iron agents, such as the new formulation ferrous bisglycinate.

## 6. Conclusion

The efficacy of Shengxuening combined with conventional iron supplementation in the treatment of anemia in pregnancy was significantly better than that of iron supplementation alone, and the adverse effects were significantly reduced compared with the control group. All anemia indexes and iron metabolism indexes were also significantly improved compared with the control group.

In conclusion, the combination of Shengxuening with conventional iron supplementation is more helpful for the treatment and recovery of patients with anemia during pregnancy, shortening the dosing time of conventional iron supplementation, reducing adverse effects, and improving patient compliance, and is recommended for clinical promotion.

## Figures and Tables

**Table 1:** Characteristics of included studies.

Study	Baseline	N (T/C)	Age (Y)		Pregnancy range (weeks)		Intervention		Duration (weeks)	Outcomes	Adverse events
			T	C	T	C	T	C			
Gu 2019 [5]	Comparable	50/50	30.1 ± 2.3	30.1 ± 2.3	28.3 ± 1.5	28.3 ± 1.5	FST	FST + SXN	4	a, b, f, h	During the experiment, no serious adverse drug reactions were observed in both groups

Li ZH 2018 [6]	Comparable	40/40	27.5 ± 2.8	27.9 ± 3.1	22.4 ± 6.3	22.0 ± 5.9	FST	FST + SXN	4	a, c	The incidence of adverse reactions in the control group was higher than that in the treatment group, and the difference was significant

Yang 2021 [7]	Comparable	80/80	29.0 ± 4.5	28.7 ± 4.1	22.6 ± 2.4	22.7 ± 2.6	FST	FST + SXN	4	a, b, e, h	During the experiment, no serious adverse drug reactions were observed in both groups

Zhang 2020 [8]	Comparable	52/52	27.12 ± 3.83	26.12 ± 4.25	31.11 ± 3.46	30.22 ± 2.83	FST	FST + SXN	4	a, b, e, h	The incidence of adverse reactions in the control group was higher than that in the treatment group, and the difference was significant

Liu 2019 [9]	Comparable	45/45	28.41 ± 5.01	28.34 ± 5.27	8.39 ± 2.39	8.3 l ± 2.34	FST	FST + SXN	12	a, b, c, d, f, g, h	NA

Lin 2018 [10]	Comparable	46/46	32.81 ± 8.04	32.83 ± 7.64	25.93 ± 3.03	25.81 ± 3.14	FST	FST + SXN	NA	a, b, e, f, h	NA

Sun 2020 [11]	Comparable	33/34	28.93 ± 3.86	29.01 ± 3.92	23.53 ± 3.47	23.61 ± 3.51	IPO	IPO + SXN	4	a, b, c, d, e, f, g, h	The incidence of adverse reactions such as stomach pain, epigastric discomfort, constipation, nausea, rash, and other adverse reactions were compared between the two groups

Liu 2020 [12]	Comparable	48/48	26.85 ± 3.35	27.12 ± 3.51	22.91 ± 2.37	23.07 ± 2.49	PIC	PIC + SXN	4	a, b, c, d, e, f, h	NA

Chen 2014 [13]	Comparable	42/42	26.1 ± 3.2	25.2 ± 2.1	20.5 ± 2.5	19.4 ± 2.2	CFSFAT	CFSFAT + SXN	4	a, b, c, d, f, g, h	The incidence of adverse reactions in the control group was higher than that in the treatment group, and the difference was significant

Zhao 2016 [14]	Comparable	40/40	29.0 ± 2.O	29.5 ± 1.5	21.0 ± 1.5	20.5 ± 2.0	CFSFAT	CFSFAT + SXN	4	a, b, c, d, f, g, h	NA

Chen 2021 [15]	Comparable	44/44	27.51 ± 3.69	27.59 ± 3.75	29.58 ± 1.27	29.64 ± 1.32	FST	FST + SXN	4	a, b, c, e, f, h	NA

Ruan 2018 [16]	Comparable	100/100	26.52 ± 2.74	26.14 ± 2.76	32.41 ± 2.61	31.86 ± 2.17	FST	FST + SXN	4	a, b, c, d, e, f, g, h	NA

Yin 2015 [17]	Comparable	85/85	28.03 ± 5.68	27.91 ± 5.73	22.74 ± 7.05	22.58 ± 6.37	FST	FST + SXN	4	a, b, c, d, e, f, g, h	NA

Fei 2021 [18]	Comparable	27/27	31.62 ± 3.86	31.61 ± 3.89	27.22 ± 5.84	27.69 ± 5.29	FL	FL + SXN	4	a, c, d, e, f, g, h	NA

Fu 2020 [19]	Comparable	39/39	25.3 ± 5.9	25.8 ± 5.2	21.0 ± 4.8	21.0 ± 4.8	DID	DID + SXN	4	a, b, c, d, e, f, g, h	The incidence of adverse reactions in the control group was higher than that in the treatment group, and the difference was significant

Liu 2016 [20]	Comparable	50/48	26.44 ± 4.58	26.89 ± 4.62	22.86 ± 3.88	23.03 ± 3.94	PIC	PIC + SXN	4	a, b, c, e, f, h	The incidence of adverse reactions in the control group was higher than that in the treatment group, and the difference was significant

Jiao 2019 [21]	Comparable	50/50	26.74 ± 2.59	26.52 ± 2.78	33.41 ± 2.77	31.82 ± 2.23	FST	FST + SXN	4	a, b, c, d, e, f, g, h	NA

“Ferrous succinate tablets = FST; Iron Protein succinylate oral solution = IPO; polysaccharide iron compound capsules = PIC; compound ferrous sulfate and folic acid tablets (CFSFAT); ferrous lactate = FL; dextran iron dispersible tablet = DID; Shengxuening tablets = SXN”. a: Hb b: RBC c: MCV d: MCHC e: SI f: TSAT g: MCHC h: clinical efficacy.

## Data Availability

The data used to support the findings of this study are included in the article.
